# Progress in Surgery

**Published:** 1903-08-22

**Authors:** 


### Progress in Surgery.

### GENITO-URINARY.

**Operation for Rupture of the Kidney.**—Though the majority of cases of rupture of the kidney recover without operation, yet occasionally, where the injury is extensive and the extravasation of blood and urine great, operation is called for. A. L. Mathieson[1] has recorded a case in which he successfully operated. A man aged 36 collided forcibly with the handle-bar of a lawn roller when moving backwards to catch a cricket ball. Six hours later he passed bloody urine and next day had great pain in the injured right kidney region, and on the second day he became feverish, had a brown dry tongue, excessive pain, and retention of urine. A lumbar incision was made, a large quantity of clotted blood cleared out, and the kidney brought out on to the loin. The organ presented two large wounds, one running transversely outwards from the hilum, almost bisecting it, and the other crossing the upper segment obliquely. Both wounds were filled with clotted blood which separated the edges to the extent of three-quarters of an inch. The ureter and peritoneum were uninjured. The wounds were cleared of blood clot and sutured with a dozen or more catgut sutures. The urine was free from blood after the fourth day, and the wound was soundly healed at the end of three weeks. Nephrectomy is rarely called for in such cases, as the kidney can recover from extensive injuries.

**Stone in the Kidney.**—This was the subject W. R. Clement Lucas selected for the Hunterian lecture of this year.[2] The following are some of the points brought out by the lecturer. *Pain* is often the symptom which first draws attention to the condition. The inconstancy, radiation, reflection and tendency to cause aching of the other organ diminish its trustworthiness as a sign upon which to base exact diagnosis. In a certain number of cases pain is altogether absent. Many post-mortem records prove this. The variability of the symptom seems to depend upon the position and degree of mobility of the stone. The secreting tissue of the kidney seems to possess little sensibility. This has been shown by absence of pain in puncturing the organ when exposed in the loin through accident or at operation and with the patient only partially under an anæsthetic. But the pelvis of the kidney and its branches are acutely sensitive. A moveable stone impinging on the outlet of the pelvis causes agonising pain. A large fixed stone may cause merely aching pain. To be of service for the diagnosis of renal stone the pain must be unilateral. Sometimes with a stone embedded in one kidney the pain is a general backache. The pain of kidney stone seems usually to be reflected along the last dorsal nerve so that in front it is felt at a lower level than behind, generally below the level of the umbilicus. A stone which has been driven into the orifice leading from a calyx of the kidney may cause renal colic. Frequent micturition and reflection of the pain to the end of the penis seem to be more marked when the stone is in the ureter than when it is in the pelvis of the kidney. The intimate connections of the renal plexus with surrounding nerves explain the occasional reflection of pain to the thigh, knee, hip, ankle and foot. The author alludes to a case which had been treated as one of sciatica, and another which had been treated by strapping the testicle owing to the concentration of the pain in that organ. Sometimes there is increased pain at night, generally in cases where there is a large stone or there are multiple stones, and this is perhaps explainable as the result of increased congestion of the kidney in the recumbent posture. Often patients cannot lie on the opposite side to the affected one. The increase of pain from jolting exercise may help in diagnosis. The lecturer described a new test for renal stone he had devised—the stamping test. The patient leans with one hand on a support, flexes the thigh on the suspected side as high as possible, and then brings the limb down again, stamping the heel firmly on the ground. A sudden acute pain is caused, the kidney having suddenly lost the support of the contracted psoas muscle and being jarred at the same time.

*Hæmaturia* may be entirely absent throughout, and curiously in such cases the stone has often been large and irregular. On the other hand, hæmaturia may be profuse and frequent and yet pain be absent. Colic frequently occurs without hæmaturia and *vice versâ*.

*Frequent micturition* is not a constant symptom, and when it does occur is most often secondary to such conditions as hæmaturia, pyuria, or excess of mucus in the urine, produced by the stone. Extreme bladder irritability and pain in the penis and perineum on micturition may be due to a stone impacted in the ureter. Thus frequency of micturition, when due to reflex irritation, is more likely to be due to a stone in the ureter or pelvis of the kidney than to one in the secreting tissue. On three occasions the author felt the grating of multiple calculi on palpating the kidney. The author's axiom for students that "pain in excess of pus indicates stone, pus in excess of pain tuberculosis pyelitis" is worth remembering.

[1] Quarterly Med. Jour., Feb., 1903, p. 134. [2] Lancet, April 25, 1903, p. 1143.

*(To be Continued.)*

